# Bio- and Biomimetic Receptors for Electrochemical Sensing of Heavy Metal Ions

**DOI:** 10.3390/s20236800

**Published:** 2020-11-28

**Authors:** Angela Maria Stortini, Maria Antonietta Baldo, Giulia Moro, Federico Polo, Ligia Maria Moretto

**Affiliations:** Department of Molecular Science and Nanosystems, Ca’ Foscari University of Venice, Via Torino 155, 30172 Venice, Italy; stortini@unive.it (A.M.S.); toni@unive.it (M.A.B.); giulia.moro@unive.it (G.M.); federico.polo@unive.it (F.P.)

**Keywords:** heavy metal ions, electrochemical biosensors, bioreceptor, biomimetic

## Abstract

Heavy metals ions (HMI), if not properly handled, used and disposed, are a hazard for the ecosystem and pose serious risks for human health. They are counted among the most common environmental pollutants, mainly originating from anthropogenic sources, such as agricultural, industrial and/or domestic effluents, atmospheric emissions, etc. To face this issue, it is necessary not only to determine the origin, distribution and the concentration of HMI but also to rapidly (possibly in real-time) monitor their concentration levels in situ. Therefore, portable, low-cost and high performing analytical tools are urgently needed. Even though in the last decades many analytical tools and methodologies have been designed to this aim, there are still several open challenges. Compared with the traditional analytical techniques, such as atomic absorption/emission spectroscopy, inductively coupled plasma mass spectrometry and/or high-performance liquid chromatography coupled with electrochemical or UV–VIS detectors, bio- and biomimetic electrochemical sensors provide high sensitivity, selectivity and rapid responses within portable and user-friendly devices. In this review, the advances in HMI sensing in the last five years (2016–2020) are addressed. Key examples of bio and biomimetic electrochemical, impedimetric and electrochemiluminescence-based sensors for Hg^2+^, Cu^2+^, Pb^2+^, Cd^2+^, Cr^6+^, Zn^2+^ and Tl^+^ are described and discussed.

## 1. Introduction

Bio- and biomimetic electrochemical sensors for heavy metal ions (HMI) monitoring have experienced a wide development in the last decade [1,2,3,4]. This broad class of analytical devices includes all sensors that can provide a quantitative or semi-quantitative detection of the target ions using a biological or biomimetic recognition element (i.e., enzyme, antibody, aptamer and molecularly imprinted polymer), which is in direct spatial contact with the electrode surface [5]. In presence of the target, the immobilized bio- or biomimetic receptor allows the detection of a change at a localized surface limiting the challenges of monitoring small-molecules in solution. Owing to their high sensitivity, selectivity and low-cost, biosensor-based analytical devices can be exploited in-situ to monitor several environmental contaminants [6,7,8]. The increasing interest in this type of sensors have been driven by concurrent factors: from the identification of suitable bio- and biomimetic receptors and the study of their interaction mechanisms to the improved design and performances of screen-printed electrodes (SPE), miniaturized devices and portable potentiostats [9,10,11].

Despite the promising performance of these analytical tools, the routinely control of HMI levels in environmental and food samples is still an issue. Indeed, HMI represent a large class of compounds, including all the elements with atomic weight higher than 63.5 g/mol and a specific gravity larger than 5 g/cm^3^, capable of undergoing several biological pathways, which lead to toxic effects for animals and human beings, and causing long-term impact on the ecosystem [12,13]. HMI such as mercury, copper, lead, cadmium, chromium, thallium, zinc, among others, are considered environmental contaminants in every respect [14,15]. Therefore, their maximum concentration levels have been strictly regulated by the international agencies, particularly the European Medicines Agency (EMA), the World Health Organization (WHO), European Environment Agency (EEA), and the US Environmental Protection Agency (EPA). An overview of the updated maximum levels for HMI in different matrices is reported in Table 1. The rising awareness about HMI harmful effects on human health is leading to the redefinition of the maximum concentration limits for these pollutants and to a continuous need to update the regulation parameters. This is the case of the new European Drinking Water Directive (DWD) updated and approved in October 2020 [16]. Therefore, the values reported in Table 1 need to be evaluated critically considering the geographical and socio-economical context of interest as well as the updates of the competent environmental agencies or prevention institutions. The performances of the sensing strategies here discussed will be evaluated in consideration of their compliance with the regulation of the country of interest.

Following the evolution in HMI regulation is crucial to design applicable sensing strategies for HMI monitoring. According to García-Miranda Ferrari et al. [10] the rate of published works in HMI sensing platforms design based on a realistic approach (matching regulation requirements, user-friendliness and cost-effectiveness) is still relatively low. Therefore, to contribute to stimulating a critical sensor design and rise the attention of researches, we propose here a wider overview of HMI sensors evolution in the last five years (2016–2020).

Instrumental techniques, such as electrothermal (ETAAS) or flame atomic absorption or emission spectroscopy (FAAS or FEAS), inductively coupled plasma mass spectroscopy (ICP-MS), high performance liquid chromatography (HPLC) coupled with electrochemical or UV-Vis-detectors, have been traditionally applied in HMI monitoring plans [24,25]. However, the highly selective and sensitive interactions of HMI with bio- and biomimetic receptors allowed them to reach comparable performances and were combined within different types of transducers, from optical to electrochemical. Electrochemical sensors are powerful sensing tools for direct and indirect monitoring of various classes of environmental contaminants, as we recently discussed [26,27]. These analytical tools have numerous advantages such as ease of use, accuracy and sensitivity, low cost, remarkable effectiveness in the multiplexed detection and on-site detection ability [28]. The user-friendly nature and requirement of simple and not expensive instrumentation make electrochemical sensing superior to spectroscopic techniques. Moreover, integration of electrochemical devices in automatic fluidic structures for a wide range of HMIs monitoring is gaining continued attention [29]. The sensitivity and selectivity of the electrochemical sensing platform can be further improved by chemical modification of bare electrodes with efficient electron mediators. The large surface area, the potential of modifications and extraordinary quantum mechanical properties of nanomaterials make them good electron mediators and promising material for electrode modification.

In this work, a critical overview of the advances in HMI electrochemical bio- and biomimetic sensors is reported providing key examples for each of the HMI listed in Table 2. In Section 2, the main classes of bio- and biomimetic receptors will be described to provide some general guidelines, while Section 3 is focused on meaningful examples of HMI sensors sorted by ion.

## 2. Bio- and Biomimetic Receptors for HMI

**Figure 1:**
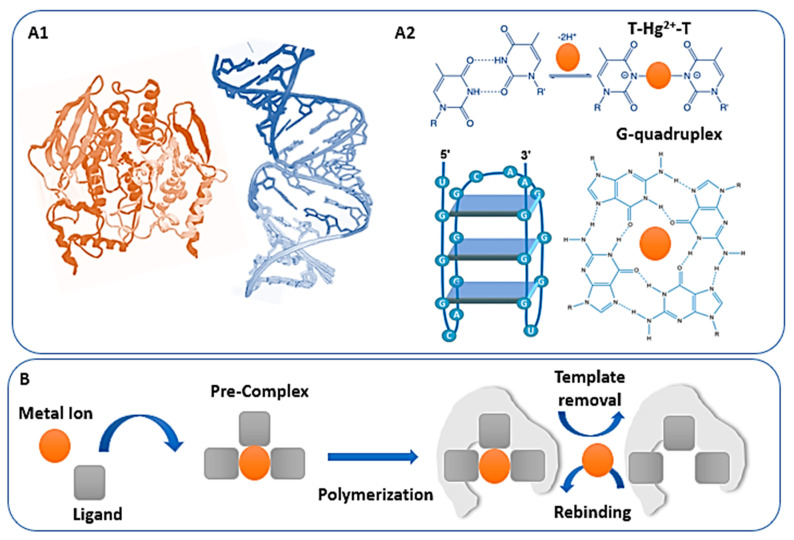
Examples of the main classes of bioreceptors discussed: protein-based bioreceptors (**A**) and ion imprinted polymers (**B**). On one side, peptides, enzymes and functional nucleic acids can be visualized through their crystallographic structures ((**A1**), examples of enzyme in orange and a DNA strand in blue) and undergo specific recognition mechanism in presence of certain HMI ((**A2**), T-Hg^2+-^T and G-quadruplex structure). On the other side, biomimetic receptors synthesis and operation mechanism can be summarized in a few fundamental steps (**B**) pre-complex formation, polymerization, template removal and rebinding).

In a sensing platform, the recognition layer enables to: recognize the analyte, pre-concentrate it at the transducer surface while limiting matrix interferences and/or undergoing processes easily detectable (i.e., conformation changes). Bio- and biomimetic receptors assure a highly selective molecular recognition taking advantage from well-known biochemical mechanisms, such as the key–lock interaction of enzymes [30,31]. So far, enzymes, proteins, antibodies, functional nucleic acids, molecularly and ion imprinted polymers receptors were successfully applied in electrochemical, electrochemiluminescence-based and impedimetric sensing strategies. To better understand the limits and potential of their applicability, an in-depth investigation in their structural features, biochemical or synthetic nature is required. The receptors synthesis, purification and integration with the transducer or nanomaterials surfaces contribute to determine the sensors performance. To develop reproducible, stabile and cost-affordable HMI sensors, the control and careful design of these steps is crucial. In Figure 1, two main classes of receptors are presented: protein-based (peptides, enzymes and functional nucleic acids, Section 2.1) and biomimetic (ion imprinted polymers, Section 2.2).

### 2.1. Peptides, Enzymes and Functional Nucleic Acids

In the recent years, different classes of protein-based receptors, exemplified by the structures in Figure 1(A1), showed promising results in HMI electrochemical sensing. Especially peptides and functional nucleic acids played a key role in the design of novel high-performing devices, while enzymes were combined within complex architectures to further improve the performance of existing sensors or used in amplification strategies [32]. Peptides are short amino acids chains (2–50 amino acids) naturally occurring and involved in different biological activities, such as metal ion homeostasis and detoxification (see [33]). Peptides applicability in HMI sensing relies on their capability to give cooperative metal-ligand interactions, involving the sulfur, nitrogen and/or oxygen atoms in their amino acid chains [34]. Enzymes are proteins acting as catalysts, and their activity might depend also on the binding of coenzymes or cofactors, such as metal ions. HMI capability to bind enzymes can not only contribute to enzyme activity but also results in inhibition mechanisms largely applied in biosensing design [35,36]. Artificial functional nucleic acids (FNA) have been preferred to antibodies and enzymes for their improved stability and the possibility to be integrated with several detection strategies. According to Liu et al. [37], FNA comprises all the nucleic acids whose functions differ from the conventional genetic ones. For the detection of HMI, the most used FNA are aptamers, metal-specific oligonucleotides (MSO) and DNAzymes [38]. These bioreceptors can be selected using SELEX (systematic evolution of ligands by exponential amplification) or in vitro selection strategies [39,40,41] aimed to the identification of the nucleic acid sequences with the higher binding affinity for the target. With respect to aptamers, the interaction with the target leads to a conformational change. These bioreceptors consist of artificial short single-stranded DNA (ssDNA) or RNA sequences that fold in specific secondary and tertiary structures when the binding event occurs. Highly performing HMI aptamers were developed for Hg^2+^, Pd^2+^, Cd^2+^ and Zn^2+^ monitoring [42,43,44]. For MSO, the presence of the target HMI causes the formation of strong metal-base complexes. The design of these specific nucleic acid sequences had followed the discovery of the specific coordination ability of Hg^2+^ and Ag^+^ ions for ssDNA rich in thyme (T-rich) and cytosine (C-rich), respectively, as described in 2015 [45,46]. Their capability to intercalate the mismatches forming T-Hg^2+^-T (see Figure 1(A2)) and C-Ag^+^-C stable pairs was used in numerous biosensing strategies mainly based on the detection of hybridization events [47,48]. The strong conformational changes caused by the ability of guanine rich ssDNA (G-rich) to self-assembly into G-quadruplex stable structures in presence of HMI, particularly Pb^2+^, as showed in Figure 1(A2) [49], allowed using also G-rich DNA-probes as bioreceptors. MSO were found to be adaptable electrode modifiers and combined within numerous indirect detection strategies. For DNAzymes, that are artificial deoxyribonucleic acid enzymes, the presence of the target HMI modifies the catalytic activity, particularly its kinetics. HMI can work as inhibitors or enhance the enzyme activity acting as co-factors, and we can take advantage from these effect in biosensor design [50,51].

### 2.2. Imprinted Polymers

Biomimetic recognition layers, such as ion or molecularly imprinted polymers (IIP or MIP, respectively) stand out in the last decade because of their ease of synthesis and integration within various electrochemical sensing strategies for HMI [52,53,54,55]. By mimicking the binding sites of biological entities, imprinted materials enable to entrap the target ion/complex or molecule by means of complexation equilibria, covalent and non-covalent interactions. IIP with their target-mimetic cavities can be synthesized in bulk, immobilized on the surface of magnetic beats, integrated directly with the electrode surface through electropolymerization, etc. [56,57,58]. Notwithstanding these numerous design options, IIP preparation relies on few common steps, summarized in Figure 1B. Firstly, the target/template ion is mixed with a polymerizable ligand to form a pre-complex (*pre-complex formation step)*. Secondly, the polymeric network is shaped preserving the target-monomer complex (*polymerization step*). Thirdly, the target/template ions are removed from the network (*template removal step*), creating high affinity sites suitable for the target ion rebinding (*rebinding step*) [55]. Depending on the format of the IIP and the design of the sensing strategy, the rebinding step can help in the preconcentration and/or detection of the target ion. As suggested by the numerous examples in Table 2, IIP are extremely versatile modifiers: They have been coupled with enzymatic amplification strategies or nanocomposites materials with synergic effects and improved performance in terms of sensitivity [59].

Apart from these classes of bio- and biomimetic receptors, the recent literature reports several examples of biosensors based on microbial fuel cells (MCF) technology, as discussed in the next section. The distribution of the bio- and biomimetic receptors here presented over the publications discussed in the next sections is summarized in Table 2 and organized per element as in the following part of the text.

## 3. HMI Sensor Overview

### 3.1. Mercury

Mercury represents a significant risk to both environment and human health globally [108]. Once it is released in the environment, it can circulate for a thousand years [109] as is (e.g., as vapors or in its elemental liquid form) or, upon chemical reactions, as its organometallic form (e.g., methylmercury (II)), whose general chemical formula is [Hg(CH_3_)]X, where X can be an halide or inorganic compounds. This phenomenon is also known as the “global mercury cycle” [110].

To date, following the *Minamata Convention on Mercury* of 2013, more than 100 Countries worldwide are taking action in the monitoring of mercury emission and its quantification. As many other toxic substances, mercury and its derivatives cause dose-dependent effects and severely affect the brain and nervous system, lungs, kidneys [111,112], developing embryos [113], heart [114], vision and hearing diseases [115]. Therefore, monitoring and precisely and timely quantifying this element, its ionic and organometallic forms (i.e., MeHg), are of great interest and a high priority worldwide.

Most of the recently developed biosensing platforms aimed at detecting Hg^2+^ are based on oligonucleotides as receptors that make the analyte available in proximity of the electrode surface. Afterwards, the analyte is detected either directly by its electroreduction (pre-concentration step), followed by its electrooxidation, or indirectly by employing a redox probe (e.g., ferrocene, thionine or tris(2,2′-bipyridyl)ruthenium(II)) whose electrooxidation provides a current or a photocurrent (electrogenerated chemiluminescence). These platforms also use nanostructured architectures (e.g., gold nanoparticles/nanorods, reduced graphene oxide, etc.) to enhance the electron transfer rate and thus improve the sensitivity.

**Figure 2:**
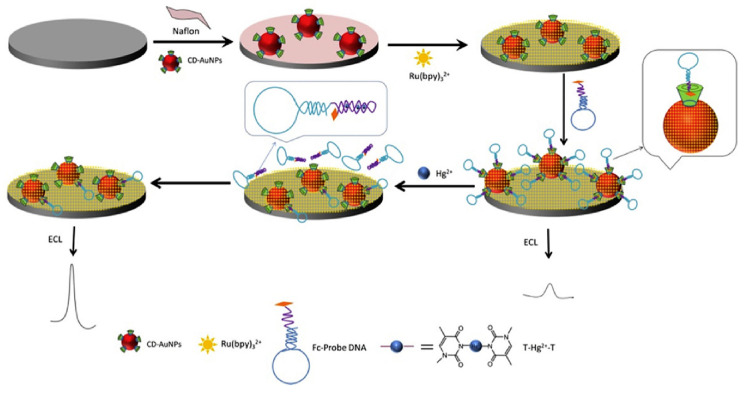
Schematic illustration of the fabrication process and sensing mechanism of the electrogenerated chemiluminescence biosensor for Hg^2+^ determination designed by Cheng et al. From the top left: modification of the glassy carbon electrode surface with Cyclodextrins-Au nanoparticles (CD-AuNps)/Nafion and Ruthenium(II) tris-(bipyridine)(Ru(bpy)_3_^2+^) followed by the immobilization of the ferrocene labelled DNA probe and, on the bottom left, the changes in presence of the target and the signal generation. Reported with permission from [62].

Taking advantage of the unique property of Hg^2+^ to bind thymine base (T) residue that leads to the formation of a T-Hg-T complex, Cheng et al. [62] devised a biosensing platform based on a hairpin-DNA probe carrying a ferrocene (Fc) moiety, as shown by the scheme in Figure 2. Fc allowed the DNA probe to bind into cyclodextrins-modified gold nanoparticles dispersed over a glassy carbon electrode, previously treated with Nafion and tris(2,2′-bipyridyl) ruthenium (II) (Ru(bpy)_3_^2+^) as the redox label. The presence of Fc close to the electrode surface and Ru(bpy)_3_^2+^ hampered the electrogenerated chemiluminescence (ECL) of the redox label (turn-off mode). However, once Hg^2+^ is present in solution, it strongly binds to the hairpin-DNA probe, which leaves the cyclodextrin thus removing Fc from the electrode and recovering the ECL signal (turn-on mode). This signal increases with increasing the amount of Hg^2+^. The biosensing platform showed a remarkable limit of detection (LOD) of 0.1 nM.

In another recent work by Ma et al. [72], a glassy carbon electrode is modified with a DNA strand that is made pairing with complementary strand carrying a Ru(bpy)_3_^2+^ redox label in a three-way DNA junction configuration. Such structure allowed the redox label to be close to the electrode surface and to emit intense light upon ECL activation. However, in the presence of Hg^2+^, the three-way DNA junction configuration changes dramatically so that the Ru(bpy)_3_^2+^ is moved away from the electrode, hampering significantly the ECL emission (turn-off mode). The ECL decreased as the concentration of Hg^2+^ in solution increased in the range from 0.10 to 10 pM with a LOD of 0.040 pM.

The possibility to determine the target ion in the attomolar range of concentrations by a DNA modified electrochemical sensor was also reported [73]. To monitor Hg^2+^ in this concentration range, Hasanjani et al. employed a pencil graphite electrode (PEG) modified with DNA/poly L-methionine gold nanoparticles, detecting Hg^2+^ via square wave anodic stripping voltammetry (SWASV). The biosensing platform is exposed to a solution containing the analyte and is biased at 0 V for 250 s, while the Hg^2+^ strongly binds to thymine base (T) residues to form again T-Hg^2+^-T complex. Afterwards the potential is scanned from −0.50 to +0.60 V at 20 Hz, causing the reduction and reoxidation of Hg^2+^ while the SWASV is recorded providing the detection of the analyte in the concentration range of 0.1 aM to 0.1 nM with an outstanding LOD of 0.004 aM.

Very recently the performances of Hg^2+^ electrochemical biosensors have been further improved also by designing new amplification strategies and combing these bioreceptors with various nano- and nanocomposite materials [116], from gold nanorods functionalized with graphene oxide [64] to porous silicon nanowires [65]. For instance, Jin et al. employed a T-rich thiolated DNA (S1), which was self-assembled on a gold electrode, and a T-rich biotin-DNA (biotin-S2) to capture Hg^2+^ in water through T-Hg-T complex formation, thus leading to a sandwich-like biosensing platform [64]. As a final step the platform was incubated with the labeling system consisting in reduced graphene oxide (RGO) functionalized with gold nanorods (AuNRs), which were then loaded with thionine (TH) and streptavidin (SA). Using differential pulse voltammetry (DPV), the TH residue present in the label RGO@AuNR-TH-SA was detected at a potential value of −0.208 V while scanning the potential from −0.5 to 0 V (vs Ag/AgCl sat reference electrode). The intensity of the peak current is proportional to the amount of TH and indirectly to the amount of Hg^2+^. Therefore, it was possible to determine the amount of analyte in the concentration range 1–200 nM with a LOD of 0.24 nM. Apart from AuNP, RGO and AuNR-based nanostructured modifiers, also metal-organic frameworks (MOF) showed a high compatibility with Hg^2+^ electrochemical sensing. Cu- and Ca-MOF are known to be excellent sorbents for mercury ions thanks to their capability to host (and not substitute) Hg^2+^ and stabilize its positive charges by deprotonation, as described by Kokkinos et al. [117]. Zhang et al. [61] used Cu-MOF/DNA probes to extract Hg^2+^ from complex matrices, such as milk. The formation of the T-Hg-T complex assured a selective recognition. The Cu-MOF/DNA/Hg^2+^ was immobilized at an AuNP modified glassy carbon electrode surface by means of a complementary DNA strand. Then, Cu^2+^ was directly detected deducing Hg^2+^ concentration, with a LOD of 4.9 fM.

### 3.2. Copper

Sources of copper are both anthropogenic, coming from forestry and mining, fossil fuel, pesticides, paints antifouling, coating boats, etc., and natural, mainly by dust re-suspension and transport, and soil erosion. In humans, the exposure to copper excess (i.e., ingestion of quantities >30 mg/L) was related to neurological disorders, such as Wilson and Alzheimer’s disease [118,119], oxidative damages [120] as well as chronic liver diseases [121].

**Figure 3:**
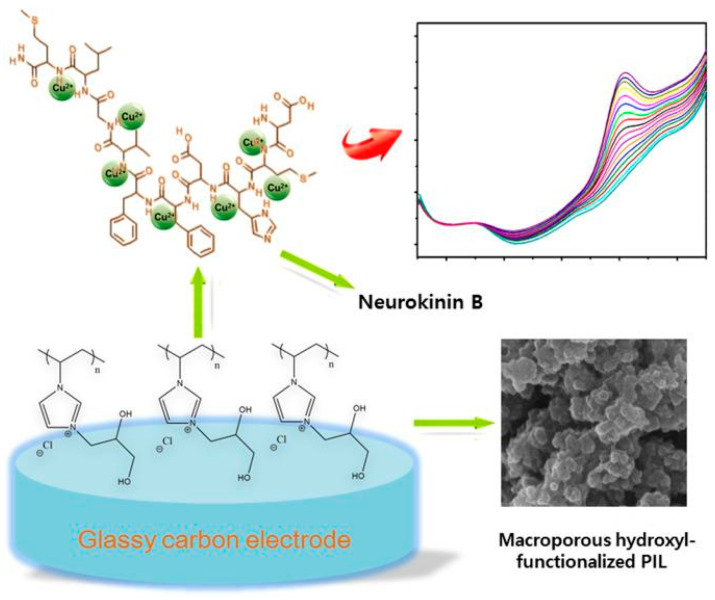
Scheme of the surface modification and Cu^2+^ detection mechanism. The surface of a glassy carbon electrode is modified with poly (ionic liquid) to form a microporous structure able to host neurokinin B. This neuropeptide binds the analyte ions and allows its determination through voltammetry. Reported with permission from [76].

The electrochemical sensing strategies published in the last five years for Cu^2+^ monitoring offer examples of most of the bio- and biomimetic recognition layers previously presented (Section 2): from peptides, to DNAzymes and imprinted polymers-based ones. In 2016, Yu et al. [76] reported a voltammetric biosensor using the neuropeptide neurokinin B (NK) as a biorecognition layer. NK was loaded at glassy carbon electrodes modified with dual hydroxyl-functionalized poly (ionic liquid), which acted as a macroporous catalyst support, as showed in Figure 3. The surface modification was finalized by electrostatic immobilization of the redox mediator, 2,2′-Azinobis-(3-ethylbenzthiazoline-6-sulfonate) and stabilized by treatment with glutaraldehyde. The electrochemical signal changes after incubation of Cu^2+^ solutions at different concentrations were followed by DPV. The analyte was determined by normalizing the current intensity of Cu^2+^ reduction peak to that of the redox mediator, acting as an inner reference. This strategy allowed to reach a LOD of 0.24 μM with a linear range between 0.9 and 36.1 μM. The authors further implemented the NK-Cu sensing strategy to enable the simultaneous determination of Cu^2+^ and β-amyloid peptide [77]. This peptide is known to undergo Cu-driven aggregation, a phenomenon directly linked to the development of Alzheimer’s disease. This example shows how HMI monitoring can be extend and combined with point-of-care applications and medical screening.

A single-HMI detection was achieved by Tian et al. [80] with a DNAzyme sensor based on a 3D ordered macroporous chitosan-Prussian blue-single walled carbon nanotubes (3DOM CS-PB-SWCNTs) nanoparticle composite. This composite served as a substrate for the immobilization of the complementary DNA strand that hybridizes with the DNA probe able to selectively bound Cu^2+^. To enhance the sensitivity, an additional amplification step was introduced by crosslinking the DNA probe with a graphene oxide–gold nanorod composite loaded with glucose oxidase and horseradish peroxidase. These enzymes are well-known to act in cascade in the glucose oxidation reaction [122]. Therefore, the electrochemical sensor response was then followed in presence of glucose: the current variations of glucose signal were linearly correlated to the logarithm of Cu^2+^ concentration. Despite its high-performance (LOD ≅10^−19^ M), the biosensor presents a complex architecture that required numerous optimization steps and seems difficult to combine with large-scale production.

A slightly simpler architecture based on an RNA-cleaving DNAzyme based biosensors for Cu^2+^/Hg^2+^ detection was recently reported [68]. The impedimetric platform was developed by functionalizing single carbon nanotubes-modified field-effect transistors (SWNTs/FET) with Cu^2+^/Hg^2+^ DNAzymes (Cuzyme and Hgzyme). When the Cuzyme binds Cu^2+^, the complementary strand is cleaved and the structural change of Cuzyme improves the conductivity of Cuzyme/SWNTs/FET biocomposite. Hg^2+^ interferences were controlled by employing a Hgzyme/SWNTs/FET in parallel and applying a Gaussian regression process to the data analysis. The authors showed how to successfully overcome a selectivity issue and design a dual detection with optimal performance (LOD ≅ 6.7 pM and linear range from 0.01 to 10^4^ nM). Real environmental samples were tested comparing ICP-MS and RNA-cleaving DNAzyme sensor outputs after extraction with relative errors ≤7%.

Wei et al. reported an IIP electrochemical sensors based on chitosan-graphene oxide composites polymer modified glassy carbon electrode (CS/GO-IIP) [81]. IIP are obtained by bulk polymerization on glassy carbon electrodes previously modified with a composite suspension CS/GO/Cu^2+^. The electrodes are then immersed in epichlorohydrin, which acts as cross linker, whilst CS and Cu^2+^ are used as complexing functional monomer and template ion, respectively. Then, EDTA chelates the template (Cu^2+^), allowing it to be removed. Once the target ion is preconcentrated at the transducer surface by the IIP, its concentration can be directly determined by pulsed voltammetric techniques, such as DPASV. A LOD value of 0.15 μM was obtained, and real samples as river and tap waters were analyzed, giving RSD% values ranging between 3.5 and 6.5%. Li et al. [82] designed a MIP-based Cu^2+^ sensors by polymerizing Methylene blue (MB) in boric acid buffer solution, using copper-glycine (Cu-Gly) complex as template. After MB-MIP polymerization the Cu-Gly complex is removed and the target-shaped cavities are occupied by HRP-Cu-Gly complex after incubation. Meanwhile, the copper in sample solutions is complexed with glycine (Cu-Gly). Once the complex is formed, a competition mechanism allows it to replace the HRP-Cu-Gly complex in the cavities. The process is observed by following the changes in the current signal of the HRP-H_2_O_2_-hydroquinone system. In optimal conditions, the sensor showed a linear range from 0.5 nM to 30 nM, and a LOD value of 42.4 pM. Cu^2+^ content in real samples such as rainwater, citric fruit juice and beer has been tested obtaining recoveries ≥95%, with RSD values ≤5% in comparison with values from ICP-MS, taken as reference method for Cu^2+^ detection.

### 3.3. Lead

**Figure 4:**
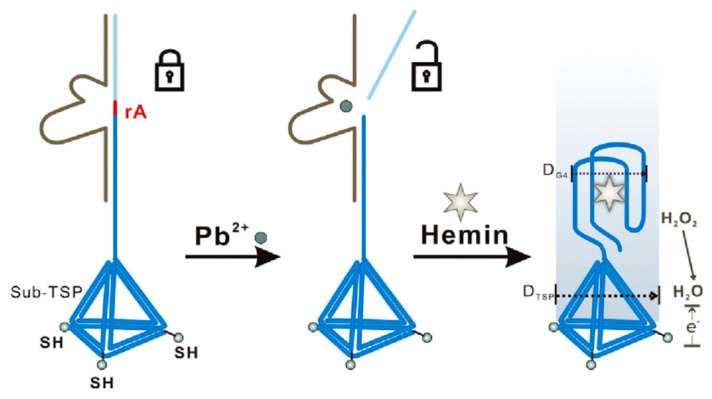
Schematic representation of the DNA tetrahedron nanostructured probes used in the label-free Pb^2+^ electrochemical biosensor by Wang et al. As reported by the authors, D_G4_ indicates the lateral dimension of hemin/G-quadruplex complex, while the D_TDP_ suggested the length of the probe base. Illustration reported with permission from [84].

For centuries, lead has been associated with man-made activities or anthropogenic sources as alloys, batteries, petrochemical industry, pigments, pesticides, bullets, glassware, mining activities, etc. Lead-related damages are primarily affecting the nervous system, reproductive apparatus and associated with hemoglobin biosynthesis disruption, alter cognitive capacities and behaviors [123]. To tackle Pb^2+^ in water and biological fluids, various biomimetic sensors were developed showing the high versatility of DNAzymes and IIP. Tan et al. [83] chose lead as a model target for the development of an immobilization free DNAzyme. The biosensor played on the electrostatic interactions occurring at the negatively charged surface of ITO electrodes. Here, the diffusion of the DNAzyme/substrate complex is limited by electrostatic repulsion. When the complex is cleaved by the presence of the target ion, the substrate labelled with a redox active probe can easily diffuse to ITO surface and be detected. This indirect detection-based sensor presents all the advantages of an immobilization free biosensing platforms, particularly suitable for portable devices development and in the technological transfer prospective. Despite the authors reporting a linear response in the range between 0.05 and 1 μM with a LOD of 0.018 μM and testing the sensor applicability on real samples, further interference studies are needed to assure the selectivity of this strategy and to evaluate the influence of the working conditions on the electrostatic interactions at stake. DNAzyme was also integrated with DNA tetrahedron probes at gold electrodes, as showed in Figure 4 [84]. Upon binding of Pb^2+^, the substrate strand forms with the hemin probe a G-quadruplex/hemin complex easily detectable through the electrocatalytic cycle occurring in presence of H_2_O_2._ This extremely elegant sensing strategy required an extensive optimization of working conditions and operational parameters, well detailed by the authors. The biosensors reached a LOD of 8 × 10^−3^ nM much lower that the Pb^2+^ concentration levels reported in Table 1.

Biomimetic sensors based on ion imprinting approach have been developed using multi-walled carbon nanotubes (MWCNT) as backbone for lead detection in real samples [85]. Bulk polymerization was carried out at vinyl functionalized carbon nanotubes (MWCNT-CH = CH_2_) in presence of the template (Pb^2+^). Comparative analysis by FTIR, XRD, TEM and EDAX techniques applied to MWCNTs, functionalized MWCNTs and MWCNTs with the imprinted layer (MWCNT-IIP) allowed to characterize the polymeric modifiers. MWCNT-IIP assessed by CV evidenced a reduction peak at −0.1 V, no detected neither for MWCNT-NIP, nor for IIP. Notwithstanding DPV voltammograms not being very clear for the quantification of the Pb^2+^ ions, the authors calculated a LOD for a concentration range of 1 to 5 ppm, corresponding to 2 × 10^−2^ μM. However, an interesting aspect of this work was the study of the nature of solute–surface interactions, and the relationship between concentrations of adsorbate and the amount of adsorbed surface at constant T value. For this purpose, Langmuir and Freundlich adsorption isotherms models for Pb^2+^ were considered. Adsorption isotherms obtained for Pb^2+^ better fit with Langmuir isotherm model, which describes a sort of monolayer adsorption and a homogeneous film. In this example, the synergic effects given by the combination of nanomaterials and IIP modifiers are clearly showed by comparing the results obtained at bare IIP, bare MWCNT and MWCNT-IIP electrodes. Checking the contribution of the single modifiers and evaluating the overall improvement obtained by their integration is fundamental to simplify and minimize the sensor architecture. This study revealed the remarkable selectivity of the material produced and its reliability to be used for the extraction of Pb^2+^ ions from various natural and industrial matrices.

A very sensitive voltammetric sensor with picomolar detection limit, based on carbon paste electrode impregnated with nano sized IIP was described by Alizadeh et al. [86]. As for the previous example, also here, the use of MWCNT allowed to improve the sensor performances showing a synergic effect with the biomimetic recognition layer. To prepare the IIP receptor, the template Pb^2+^-ITA (itaconic acid) underwent a bulk polymerization on MWCNT functionalized carbon paste electrodes. The sensor assembly IIP/MWCNT-CPE consisted of a mixture IIP (7% *w*/*w*), MWCNT (6% *w*/*w*), graphite powder (74.8% *w*/*w*) and paraffin oil (12.2% *w*/*w*) which is packed to a hole. The sensor showed a LOD value of 3.8 pM and two linear concentration ranges from 0.01 to 0.50 nM and from 1 to 80 nM. Cations as Co^2+^, Ag^+^, Ni^2+^, Cd^2+^, Hg^2+^ exhibit no significant effect on the electrode signal. In fact, for a 10 nM Pb^2+^ ion, the interference of cations as Co^2+^, Ag+, Ni^2+^, Cd^2+^ and Hg^2+^ are not significant. Instead, 50-fold excess of Fe^2+^, Zn^2+^ and 40-fold excess of Cu^2+^ influence the response of the sensor. To test its applicability to real samples, river water and seawater samples spiked with Pb^2+^ ions were analyzed, obtaining recovery values >95% and a satisfactory agreement with data found by ICP-OES reference technique.

The possibility to integrate IIP with magnetic nanobeads was tested by Ghanei-Motlagh and Taher in the development of a Pb^2+^ nano-IIP electrochemical sensor [87]. The nanobeads were first functionalized in presence of a vinyl monomer and the template (Pb (NO_3_)_2_). This complexation step was followed by the polymerization carried out adding a cross-linker and an initiator. Pb^2+^ determination was performed by DPASV, reaching a LOD value of 0.5 μg L^−1^ within a linear range between 3 and 5 μg L^−1^. Interferences from some cations were examined and quantified as <5% also for real samples, such as filtered river and tap waters.

Recently, Dali et al. [89] reported a biomass based Pb^2+^ and Cd^2+^ sensing strategies in which the ion recognition and reduction are operated by a biomass composite with SWCNT at GCE surface. Once immobilized the Pb^0^ and Cu^0^ are determined via anodic stripping. The ion recognition was achieved via the cell walls rich in chemical groups able to interact with the target ions. This type of biosensor has good performances in terms of sensitivity.

### 3.4. Cadmium

Cadmium ions reach the environment mainly from industrial processes and agricultural activities. They cause severe risks to living organisms due to their non-biodegradability and long-time persistence [124]. Based on several epidemiological and clinical studies, cadmium compounds were classified as known human carcinogens, primarily associated with lung, prostate, liver, kidney and pancreatic cancers [125,126]. Because of these harmful effects, the maximum concentration level for Cd^2+^ in drinking water established by U.S. EPA limit and recent EU guidelines is 5 μg L^−1^ [16,17,18] whereas WHO recommends 3 μg L^−1^ [20].

Enzyme–based electrochemical biosensors recently proposed for monitoring Cd^2+^ ions, in most cases, analyze simultaneously also other HMI, as Cu^2+,^ Cr^6+^ or Co^2+^. This type of sensors was mainly based on the inhibition of enzymes, such as beta galactosidase (β−gal) as proposed by Fourou et al. [91]. This work focus on the development of a selective biosensor based on the inhibition of β−gal immobilized on a bare gold electrode, as electrochemical transducer, by crosslinking with glutaraldehyde. The enzymatic reaction with the metal ion was monitored by conductometry, and the detection of Cd^2+^ was carried out by EIS and SWV measurements in the presence of [Fe(CN)_6_]^3−/4−^ redox probe in phosphate buffer solution at pH 7.4, obtaining wide linear concentration ranges, and LOD values of 2.85 × 10^−8^ and 3.22 × 10^−11^ M using EIS and SVW, respectively.

Exploiting an amperometric strategy, a biosensor based on the inhibition of the enzyme acetylcholinesterase (AChE) was described by Gumpu et al. [92]. In this work, a Pt electrode was modified firstly with Ru(II)-tris(bypiridyl)-graphene oxide (GO) nanocomposite, which promoted the next immobilization of large amount of AChE, improving the analytical performance of this inhibition based biosensor as well as preventing enzyme fouling. The results demonstrated the selective nature of such sensor assay in quantifying Cd^2+^ ions in a linear range of 0.02–0.7 μM, LOD of 0.07 μM with a fast response time. The reliability of this biosensor was proved by testing contaminated river and industrial wastewaters, with results in accordance with data found by AAS technique; however, the authors pointed out that the quite low regeneration and stability of AChE limited its application to continuous monitoring of the target HMIs. With the aim of improving the stability of the enzymatic layer immobilized onto the substrate, David et al. [90] developed and optimized a strategy to perform a stable encapsulation of the AChE enzyme, through the sol-gel method, onto carbon ink screen-printed electrodes. By this approach, the enzyme was immobilized on the electrode surface using, instead of the well-known cross-linking method with glutaraldehyde, various combinations of three sol–gel precursors, namely TEOS, TMOS and MTMOS, without the usual addition of alcohol. The sensors prepared with TEOS gave the best results, and they were employed for the amperometric detection of Cd^2+^ ions, showing a LOD value of 0.19 μg L^−1^ and promising potentialities for the development of disposable biosensors.

Among the biosensing platforms recently developed for monitoring Cd^2+^ ions, aptamers showed particularly promising results. Zhad et al. [93] designed a “signal-on” aptasensor for Cd^2+,^ using a thiolate and methylene blue (MB)-labeled aptamer immobilized on gold disk electrodes. In the absence of the target ion, the aptamer probe is partially folded. Upon Cd^2+^ binding, it changes its conformation, resulting in an increase of the MB current intensity. Alternating current voltammetry and cyclic voltammetry were applied to follow the binding, enabling to detect Cd^2+^ even in the presence of other HMI, with a linear dynamic range between 250 nM and 1 µM, and a LOD value of 92 nM. As for other folding- and dynamics-based electrochemical biosensors, multiple uses (up to three) were possible by introducing a regeneration step. To address the need of portable devices, researches focused on the development of highly sensitive aptasensors based on screen-printed electrodes (SPE) [74,94]. Li et al. [94] proposed a simple label-free aptasensor on gold SPE using the issAP08_Cd^2+^ aptamer with a thiol linker as a bioreceptor. Once Cd^2+^ ions were pre-concentrated at the SPE surface by forming the aptamer–target complex, increased peak current intensities were recorded by CV and DPV. The linear correlation between current density values and the logarithm of Cd^2+^ allowed to reach a LOD of 0.05 ng mL^−1^ with a linear concentration range from 0.1 ng mL^−1^ to 1000 ng mL^−1^ and a good selectivity in presence of other HMI, such as Cr^3+^, Hg^2+^, Pb^2+^ and Ni^2+^.

A potentiometric aptasensor array for the simultaneous detection of Cd^2+^ and Hg^2+^ was developed by Tang et al. [74]. A multichannel disposable screen-printed carbon electrode (SPCE) modified with reduced graphene oxide and dendritic nanostructured gold was used to increase the stability and the effective area of SPCE channels. By immobilizing thiolated aptamers labeled AuNPs on the SPCE channels, the target ions were recognized specifically and determined by an open circuit potential (OCP) technique. An internal calibration DNA sequence (IC-DNA) was used to provide an internal calibration potential, allowing the background influence to be subtracted. Under the optimized conditions, linear concentration range from 2.5 pM to 2.5 µM, and detection limit of 0.62 pM for detection of Cd^2+^ ions, were reported. The authors applied this array to the analysis of real water samples, i.e., tap, lake and river waters, obtaining recovery values ranging from 98.92 to 101.01%.

Another recent study on the electrochemical aptasensing for Cd^2+^ on a SPCE modified with carbon black (CB) and AuNPs was reported by Fakude et al. [95]. Upon interaction with the metal ion, the aptamer underwent a conformational change into a hairpin-like structure, allowing to the redox probe (the ferri/ferrocyanide couple) an easy access to the electrode surface, thus leading to an increase in the current intensities. The signal changes were followed by SWV, and the target was determined in the linear range of 1–50 ppb with a LOD of 0.14 ppb, showing good reproducibility, stability and excellent selectivity respect to other metal ions.

Moreover, glassy carbon electrode (GCE) modified with chitosan (CS) and AuNPs was used as the electrochemical base to design another Cd-aptamer biosensor [96]. Its analytical performance was investigated by DPV measurements, obtaining a LOD value of 0.05 nM and a wide linear range from 0.001 to 100 nM.

The possibility to improve Cd^2+^-aptasensors by including reduced graphene oxide (rGO) and graphite carbon nitride (g-C_3_N_4_) nanocomposite materials was reported by Wang et al. [97]. The authors immobilized aptamers with a carboxyl linker on a rGO/g-C_3_N_4_ nanocomposite, thus obtaining a functional biosensor whose efficient and specific interaction with Cd^2+^ ions originated from the selected sequence in aptamers and their bonding with g-C_3_N_4_. DPASV was used to characterize and evaluate the analytical performance of the biosensor, which exhibited good stability and sensitivity for Cd detection, with linear calibration curves ranging from 1 nM to 1 μM, and from 1 μM to 1 mM, and a LOD value of 0.337 nM. The applicability of this aptasensor for Cd detection in real samples was then verified in tap water, lake water and industrial waste from a paper mill.

Finally, microbial fuel cells (MFCs), using both bacterial consortia and single strains, represent another recently growing strategy for development of electro-biochemical sensors in HMIs detection, included Cd^2+^ [127]. These biosensors are based on the changes in voltage signal, through the activity of immobilized electrogenic bacteria. For instance, a dual chamber self-powered MFC-base biosensor for real-time monitoring toxicity in water containing Cd^2+^ ions, together with other metal ions, was proposed by Yu et al. [128]. The results showed that the respiration activity of electrochemically active bacteria can be inhibited in the presence of the target heavy metal ions, which can be detected by such simple, low cost and sensitive self-powered MFC-base biosensors. Analogously, a dual chamber MFC biosensor was also designed by Xie et al. for real-time and sensitive detection of Cd^2+^ and other HMIs in wastewaters quality monitoring [100].

### 3.5. Chromium

Chromium was discovered at the end of XVIII century, and it is naturally present in the Earth’s crust as chromium minerals, mostly as chromite ores. The name of chromium, from Greek “chroma”, means “color”, due to the great number of brightly colored compound of this element. For this reason, in the past, chromium minerals were mainly used to produce paints and pigments; now, the principal uses are as metal alloying agent as in stainless steel production, for chrome plating, leather tanning and other industrial processes. Due to the well-known carcinogenicity and genotoxicity of hexavalent chromium (Cr(VI)), compared to trivalent Cr(III), its use and release in the environment are of major concern and its monitoring strictly ruled [129].

Electrochemical Cr(VI) detection was successfully achieved by the enzymatic biosensor developed by Fourou et al. [91], described above for Cd^2+^ ions. Indeed, the inhibition of β-gal and the following decrease of its activity were observed also in presence of Cr(VI). The quantification of the Cr(VI) species was carried out by EIS and SWV measurements, obtaining LOD values in the order of ng/L (91.7 ng/L) and linear concentration range from 10^−2^ to 10^−4^ μg/L. Test measurements in spiked river water samples gave Cr(VI) recoveries in the range of 95–103% and RSD values <6%.

Detection of both hexavalent (Cr(VI)) and trivalent (Cr(III)) species in aqueous samples was illustrated by Prabhakaran et al. [103]. These authors proposed a microbial-based biosensor consisting of a carbon paste electrode (CPE) modified by coating its surface with a gram-negative bacterial strain of *Citrobacter freundii* (Cf-CPE), which is a suitable sorbent of some heavy metals. The CV characterization of the sensor evidenced the effects of biomass loading, scan rate and the concentration ratio of Cr(VI)/Cr(III) ions at different pH values. Moreover, DPCSV measurements gave reliable stripping voltammograms in the concentration ranges of 1 × 10^−9^ to 1 × 10^−4^ M and of 1 × 10^−7^ to 1 × 10^−2^ M, with LOD values of 1 × 10^−9^ and 1 × 10^−7^ M, for Cr(VI) and Cr(III) species, respectively. The stability and utility of the developed biosensor for the analysis of Cr(VI) and Cr(III) ions in chromite mine water samples has been also evaluated, giving RSD% values within 6% for both analytes, and well-comparable results with a spectrophotometric method taken as reference.

A green microbial fuel cell-based biosensor (MFC) for in situ Cr(VI) measurements in electroplating wastewaters, using the anaerobic bacterium *Exiguobacterium aestuarii YC211*, has been proposed by Wu et al. [104]. *YC11* is a Cr(VI)-reducing, salt-tolerant and exoelectrogenic bacterium, that are advantageous characteristics to be exploited for in situ or ex situ measurements of Cr(VI). To evaluate its feasibility, in this study, the bacterium was isolated from the electroplating wastewater and then inoculated in the anode of an MFC, and the effects of various experimental parameters such as medium concentration, NaCl content, pH, temperature and coexisting ions on the MFC performance have been investigated. Measurements in artificial wastewaters samples and real plant effluents carried out by such MFC biosensor and, for comparison, by colorimetric methods, gave well-comparable and promising results.

Finally, in a very recent work, Lazzarini Behrmann et al. [101] developed a portable self-powered microbial electrochemical biosensor for the on-line monitoring and also the simultaneous removal of Cr(VI) species. In this system, the microbial sensing unit was formed by *Pseudomonas veronii* 2E bacterial strain, which is able to biotransform and reduce Cr(VI) to Cr(III), based on Cr(VI) reductase and an external electron donor. Self-powered behavior was obtained by coupling the anodic sensing unit to a Pt-free activated carbon-based cathode, in a single-chamber microbial fuel cell (MFC). The current generated by applying a 1000-Ω resistor between anode and cathode allowed Cr(VI) monitoring with linear responses in the range 4–18.5 mg L^−1^. However, according to the authors remarks, other toxic and inhibiting compounds could affect the current response of the biosensor, thus resulting not specific for the target Cr(VI) species.

### 3.6. Zinc

Zinc is an essential trace element, or micronutrient, required for the normal physiological growth and reproduction of all living cells systems [130]. It plays a key role in gene transcription and neural signal transmission, is essential for the functionality of more than 300 enzymes and fulfills an immunological function. Toxic effects are produced only by exposure to high doses of Zn^2+^ ions making acute zinc intoxication, with cytotoxic effects particularly on the brain, even though it is a rare event [131]. In addition to acute intoxication, long-term and high dose zinc supplementation interfere with the uptake of copper, leading to symptoms due to copper deficiency as anemia and neutropenia. Considering the role of zinc in human health, the limit established by WHO for Zn^2+^ ions in drinking water is 3 mg L^−1^ [20], as reported in Table 1.

The determination of Zn^2+^ ions by electrochemical biosensors has been often proposed jointly with other HMIs, and most of the biosensing platforms recently developed have been already discussed [127,128]. For instance, dual chamber MFC-based biosensors described above for Cd^2+^ detection (see Section 3.4) [128] were capable to monitor also Zn^2+^ ions, together with several other HMIs. Moreover, recently Labro et al. [106] proposed photosynthetic MFCs (pMFCs) that use photosynthetic microorganisms, such as algae and cyanobacteria, to provide reducing power at the anode. A reproducible light-dependent electrogenic effect occurs in such bioelectrochemical system, as algae or cyanobacteria convert light to electrical energy. In this study, the authors exploited such phenomenon to construct an environmental biosensor and reported the effect of common toxicants, included Zn^2+^, on the electrogenic activity of the carbon electrode surface dwelled in benthic microalgae and cyanobacteria. A decrease in the light-dependent electrical response proportional to the metal ions concentration was found, indicating the utility of these systems based on microbial metabolism as potential environmental biosensors.

Li et al. [105] developed an electrochemical biosensor using paper-based microfluidic channels with reduced graphene oxide (rGO), chitosan and integrating hemin/G-quadruplex structure. This latter acted as HRP-mimicking DNAzyme, to enhance the catalytic properties of the sensor. The so-designed steric lab-on-paper device allowed the determination of Zn^2+^ ions with a LOD of 0.03 nM and a wide linear range (0.1–7000 nM). The current signals recorded in the presence of other seven interfering HMIs demonstrated a very high selectivity towards Zn^2+^ ions. Therefore, the biosensor resulted suitable for working in complex biological and environmental systems: Practical applications in liver cell extracts and tap water samples were reported.

**Figure 5:**
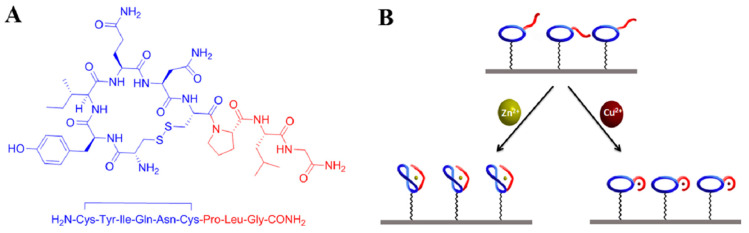
Structure of the neuropeptide used by Tadi et al. (**A**) and illustration of its behavior in presence of Zn^2+^ and Cu^2+^ ions (**B**). Reported with permission from [78].

In the last years, also the use of peptides played a key role in HMI biosensing thanks to peptides specificity in binding metal ions and their conformational changes upon the complex formation, which make them suitable bioreceptors. Tadi et al. [78] used oxytocin (OT), a neuropeptide whose activity is modulated by binding to Zn^2+,^ besides to Cu^2+^ ions, as recognition layer in the design of an impedimetric biosensor, as showed in Figure 5. The authors aimed to detect these cations in biofluids at physiological pH. The study demonstrated that the metal ions-dependent change in the conformation of OT produces unique electrochemical impedance signal patterns, leading to the selective detection of Zn^2+^ and Cu^2+^ ions, even when both ions are present in the sample. The authors then applied the so designed OT sensor to monitor the Zn/Cu ratio in diluted human sera samples of healthy control and multiple sclerosis patients, opening the way for the development of point-of-care sensing devices for biomedical research.

To implement and simplify the OT-based sensor design, Mervinejsky et al. [79] presented a further investigation about single-step formation of a native OT monolayer onto gold surface, taking advantage of the native disulfide bond of OT for anchoring the peptide to the Au electrode surface. This self-assembled OT monolayer was characterized by surface spectroscopic analysis and then applied to the impedimetric Zn^2+^ sensing, enabling the detection of Zn^2+^ ions in biofluids with a wide dynamic range from 10^−13^ to 10^−3^ M. The results obtained in this work suggested therefore that native neuropeptides can be promising as biomimetic tool for biosensing applications.

### 3.7. Thallium

Thallium is a natural component of the upper continental crust, with an estimated concentration of about 0.75 mg kg^−1^ [132]; it is mainly present as oxides or salts and dispersed at trace levels in clays; sludges and minerals of copper, lead or zinc. The major sources of thallium pollution are related to anthropogenic activities, such as mining, industrial processes and high technology applications, and from its wide use in the past as pesticide and rodenticide [133]. Thallium has been reported to have a very high toxicity, even more than Hg, for all living organisms and human health, because its compounds are more water soluble with a high likelihood to bioaccumulate [134] causing severe damages to nervous and gastrointestinal systems. Considering these effects, the maximum level for Tl^+^ content established for drinking water by US EPA must be below 2 μg L^−1^, as reported in Table 1. However, in spite of its high toxicity, thallium has been rarely studied in comparison to other toxic metals (such as Cd, Hg or Pb) [66], probably due to poor quality of the response of many classical analytical techniques to the very low concentration levels of this HMI.

Nasiri-Majd et al. [107] developed a strategy based on a carbon paste electrode modified with a nanosized thallium imprinted polymer (Tl-IP) and multiwalled carbon nanotubes (Tl-IP–MWCNT–CPE). Tl-IP enables the selective preconcentration and determination of Tl^+^ by DPASV. For the polymerization reaction, ethylene glycol dimethacrylate as the crosslinking monomer, and methacrylic acid as the functional monomer, together with the initiator 2,2′-azobis(isobutyronitrile), have been employed. In this study, the role of experimental parameters such as the electrode composition, the supporting electrolyte, the reduction potential and the accumulation time, on the performance of the Tl-IP–MWCNT–CPE sensor, has been carefully investigated. Tl^+^ content in tap water, well water, wastewater and human hair was successfully determined with recovery values ranging from 95 to 103%, linear concentration ranges between 3.0 and 240 ng mL^−1^ and LOD ≅ 0.76 ng mL^−1^.

Considering the extensive use of liquid eutectic Tl-Hg alloys in resistance and thermostatic devices, designing of sensitive analytical platforms for the detection of both these metal ions is strongly demanded in the literature. Shah et al. [66] proposed a new amino acid based electrochemical sensing for the simultaneous detection of Tl^+^ and Hg^2+^ ions based on amino acids-metal ions interactions. To this aim, the behavior of ten amino acids was evaluated after immobilization at the surface of glassy carbon electrodes. Among them, glycine (Gly) functionalized GCE gave the best response for the co-sensing of the two HMIs in water samples, and this result was related to the relatively small size of glycine, which offers minimum steric hindrance and thus more sites for metal ions accumulation with respect to amino acids with longer side chains. The capability of glycine to selectively preconcentrate analytes at the electrode surface was supported by computational findings. The performance of the designed sensing platform was tested by EIS, CV, SWASV and chronocoulometric techniques. Under optimized conditions, employing the Britton–Robinson Buffer (BRB) at pH 4 as the supporting electrolyte, the Gly-CGE modified electrode was able to detect Tl^+^ ions at sub-nanomolar concentration levels. A LOD value of 0.175 nM, well below the threshold limit set by US EPA [17], linear calibration curves from 2 nM to 0.2 mM and recoveries >95% with RSD values <5%, taken in spiked drinking, river and industrial wastewaters, were reported. Despite the limited number of meaningful examples of electrochemical biosensors for Tl^+^ monitoring, the strategies reported so far have a great potential enabling to reach the regulation limits and to operate simultaneous detection of Tl^+^ and Hg^2+^.

## 4. Conclusions and Perspectives

In this review, we discussed key examples of bio- and biomimetic electrochemical sensing strategies for HMI monitoring reported in the last five years (2016–2020). The evolution in the design of HMI showed a progressive shift towards aptamer, DNAzyme-based strategies, instead of enzymatic ones, and the rising interest in imprinted polymer recognition layers, while the role of enzymes was mainly focused on signal amplification. In most of the examples presented, nanomaterial or nanocomposites played a crucial role in the improvement of the sensor performance giving synergic effects with the recognition layer selected. Despite the good responses in terms of sensitivity, selectivity, possibility to meet the LODs indicated and real sample applicability, most of the sensing strategies discussed are not portable or compatible with portable setups so far. This lack of a realistic approach, already claimed by García-Miranda Ferrari et al. [10], needs to be urgently addressed. In this frame, this critical revision about electrochemical, electro-chemiluminescent and impedimetric HMI sensors is instrumental. In our prospective, the comparison of different classes of recognition layer plays a key role being one of the first steps of sensing strategies design and needs to be carefully analyzed. We believe that a critical revision of the recent literature, a careful design and a well-defined analytical context (i.e., identification of a target matrix, concentration levels, possible interfering agents, working conditions, etc.) can help in the design of high-performing, portable HMI sensors able to overcome the challenges of technological transfer and reach their application field.

## Figures and Tables

**Table 1:** Limits and maximum levels of heavy metals ions in drinking and wastewaters, foodstuff, drugs.

Matrix	HMI	Concentration Limit			Maximum Level	Units	Notes
		EPA [17]	EU Directis [18]	Updated EU directive 2020 [16]			
Drinking water	Cd	5	5	5	-	μg L^−1^	
	Cr	100	50	25	-		Total inorganic Cr. The updated value shall be met, at the latest, by 15 years after the day of entry into force of the new EU directive [16]
	Pb	15	10	5	-		The updated value shall be met, at the latest, by 15 years after the day of entry into force of the new EU directive [16]
	Hg	2	1	1	-		
	Cu	1300	2000	2000	-		
	Tl	2	-	-	0.002 [19]	mg L^−1^	
	Zn	-	-	-	3 [20]		
Waste water [21]	Cd		0.05			mg L^−1^	Emission limit values for discharges of wastewaters from the cleaning of waste gases [21]
	Cr		0.5				
	Cu		0.5				
	Pb		0.2				
	Hg		0.03				
	Tl		0.05				
	Zn		1.5				
Food [22] Fats and oils, milk and derivates, meat and fish products, cereals, legumes, vegetables	Pb	-	-	-	0.020 to 1.5	mg kg^−1^	
Meat, mollusks, cereals, vegetables and fruit	Cd	-	-	-	0.050 to 1.0		
Fish products	Hg	-	-	-	0.50		
Drugs [23] Products Substances Excipients	Cd	-	-	-	0.5/0.2/0.3	μg g^−1^	Oral/Parenteral/Inhalation
	Pb	-	-	-	0.5/0.5/0.5		
	Hg	-	-	-	3/0.3/0.1		
	Tl	-	-	-	0.8/0.8/0.8		
	Cu	-	-	-	300/150/1		
	Cr	-	-	-	1100/110/0.3		

**Table 2:** Electrochemical, impedimetric and electrochemiluminescence-based sensing strategies for HMI: overview of key examples from the last five years (2016–2020).

Metal Ion	Recognition Layer	Linear Range	Limit of Detection	Matrix	Reference
Hg^2+^	Polythymine peptide nucleic acid	5–500 nM	4.5 nM	tap water	[60]
	CuMOFs Thymine DNA strands (T-rich)	10 fM–100 nM	4.8 fM	pure fresh milk	[61]
	Thymine ssDNA (T-rich)	0.02–800 ng/mL	0.1 nM	lily	[62]
	Phosphorothioate oligonucleotide (PTO)	10^−11^–10^−7^ M	2.34 × 10^−11^ M	reference material	[63]
	RGO@AuNR-TH-SA Thymine	1–200 nM	0.24 nM	tap water wastewater	[64]
	Silicon nanowires Triglycine (Glyl-Gly-Gly)	10^−3^–10^−8^ M	10^−6^ M	-	[65]
	Gly modified GCE	2 nM–0.2 mM	0.23 nM	drinking water, spring water, river water, industrial wastewater	[66]
	ssDNA for signal output and nicking endonuclease assisted cycling amplification	0.01–100 nM	3 pM	river water	[67]
	Hgzyme/SWNTs/FET DNAzyme	10–10,000 nM	3.43 nM	pait, soil	[68]
	sDNA/MOF-Au	0.10 aM–100 nM	0.001 aM	fresh milk, yogurt and infant milk powder	[69]
	(APT/Au/MoS_2_-MWCNT)	0.1 nM–1 μM	0.05 nM	tap water	[70]
	Aptamer/Au/Pt@CNF/CILE	1.0 × 10^−15^–1.0 × 10^−6^ M	0.33 fM	domestic and mineral water	[71]
	Thiolated DNA strand	0.01–0.1 mg L^−1^	0.01 mg L^−1^ 0.005 mg L^−1^	water dmso (20%)	[44]
	DNA three-way junction structure (DNA-TWJ)	0.1–10 pM	0.04 pM	water pipes	[72]
	DNA/PMET-AuNPs/PGE	0.1 aM–0.1 nM	0.004 aM	sea water fish	[73]
	Aptamer	2.5 pM–2.5 μM	2.0 pM	tap water, lake water, river water	[74]
	GlyGlyGlycine-modified PSiNWs	10^−3^ –10^−9^ M	10^−7^ M	-	[65]
Cu^2+^	Glutathione modified SPE with carbon nanofiber electrode (GSH-SPCNFE)	10.1–150.1 mg L^−1^	3.0 mg L^−1^	wastewater certified reference material (ERMs-CA71)	[75]
	DHF-PIL-ABTS/NKB/Glu	0.9–36.1 μM	LOD 0.24 μM LOQ 0.6 μM	cerebrospinal fluid hippocampus	[76]
	Neurokinin B (NKB) ABTS-PDDA/CNTs-NKB	0.1–10 μM	0.04 μM	plasma hippocampus	[77]
	Oxytocin (OT)	-	500 fM	healthy and MS sera patients	[78]
	Oxytocin (OT)	10^−13^–10^−9^ M	-	-	[79]
	Cuzyme/SWNTs/FET	0,01–10,000 nM	0.0064 nM	pait, soil	[68]
	3DOM CS-PB-SWCNTs	10^−18^–10^−5^ M	10^−19^ M	river water	[80]
	CS/GO/Cu (II)	0.5–100 μ M	0.15 μM	tap water, river water	[81]
	MIECS (MIP/Cu-Gly)	0.5–30 nM	42.4 pM	running water, citric fruit juice, rainwater, beer, standard food	[82]
Pb^2+^	Glutathione modified SPE with carbon nanofiber electrode (GSH-SPCNFE)	10.8–150.1 mg L^−1^	3.2 mg L^−1^	wastewater certified reference material (ERMs-CA71)	[75]
	DNAzymes and ITO based immobilization	0.05–1 μM	0.018 μM	river water, tap water	[83]
	DNA nanostructure DNAzyme and G-quadruplex/hemin	0.01–1000 nM	0.008 nM	tap water, pool water	[84]
	MWCNT-IIP	1–5 mg L^−1^	2 × 10^−2^ µM	mining effluent, lake water, food, cosmetics	[85]
	Itaconic acid-Pb^2+^ complex and ethylene glycol dimethacrylate (IIP/MWCNT-CP)	1.0 × 10^−11^–5 × 10^−10^ M 1.0 × 10^−9^–8 × 10^−8^ M	3.8 × 10^−12^ M	seawater, river water	[86]
	IIP-MWCNTs-CPE	3–55 μg L^−1^	0.5 μg L^−1^	river water, aqueduct water, copper factory, wastewater, coal processing wastewater	[87]
	GCE modified with magnetic IIP nanoparticles (IIP-GCE)	0.1–5 ng mL^−1^ 5–80 ng mL^−1^	LOD 0.05 ng mL^−1^ LOQ 0.16 ng mL^−1^	tap water, river water, rainwater, fruit juice	[88]
	GCE functionalized with carbon nanotubes (SWCNTs-COOH) + filamentous fungi	validated	0.01 μM	unknown	[89]
Cd^2+^	Acetylcholinesterase (AChE)	2.50–25.00 mg L^−1^	0.19 mg L^−1^	river water	[90]
	Beta galactosidase enzyme (β-gal) on bare gold electrode	EIS 2.36 × 10^−3^–2.36 × 10^7^ mg L^−1^ SWV 2.36 × 10^−3^–2.94 × 10^7^ mg L^−1^	EIS 6.95 mg L^−1^ SWV 7.61 × 10^−3^ mg L^−1^	river water	[91]
	Pt/Ru(II)-tris(bipy)-GO/AChE electrode	0.02–0.7 μM	0.07 μM	river water, wastewater	[92]
	5′HS-(CH2)6-GGACTGTTGTGGTATTATTTTTGGTTGTGCAGTATG-MB 3′	250 nM–1μM	92 nM	tap water, synthetic saliva	[93]
	Aptamer issAP08-Cd	0.1–1000.0 ng mL^−1^	0.05 ng mL^−1^	fish, river water	[94]
	Aptamer	2.5 pM–2.5 μM	0.62 pM	tap water, lake water, river water	[74]
	36-base thiolated ssDNA aptamer on the SPCE-CB-AuNPs	1–50 μg L^−1^	0.14 μg L^−1^	tap water, industrial effluent	[95]
	aptamer on GCE—chitosan (CS)	0.001–100 nM (1.124 × 10⁻^13^–1.124 × 10⁻⁸ g mL^−1^)	0.04995 pM (5.614 × 10⁻^1^⁵ g mL^−1^)	tap water	[96]
	carboxyl-terminated aptamers with an appropriately regulated rGO/g-C_3_N_4_ nanocomposite	1 nM–1 μM 1 μM–1 mM	0.337 nM	tap water lake water industrial waste	[97]
	MWCNT-IIP	-	0.03 μM	lake water, pigments, cosmetics, fertilizers	[98]
	(PPy/rGO) composite for trace level determination of Cd(II)	1–100 μg L^−1^	0.26 μg L^−1^	lake water, river water	[99]
	Double-chamber MFC	0.4–10 mg L^−1^	---	wastewater	[100]
Cr^6+^	Beta galactosidase enzyme (β-gal) on bare gold electrode	EIS 2.94 × 10^−2^–2.94 × 10^4^ mg L^−1^ SWV 2.94 × 10^−2^–2.94 × 10^4^ mg L^−1^	EIS 9.17 × 10^−2^ mg L^−1^ SWV 9,17 × 10^−2^ mg L^−1^	river water	[91]
	self-powered microbial electrochemical sensor—(Pseudomonas *P. veronii* 2E)	4–18.5 mg L^−1^	2.4 mg L^−1^		[101]
	b and g-Proteobacteria	-	5 mg L^−1^		[102]
	Double-chamber MFC	0.3–10 mg L^−1^	-	wastewater	[100]
	Carbon paste electrode modified with Citrobacter freundii (Cf–CPE)	-	CV 1 × 10^−4^ M Cr(VI) 5 × 10^−4^ M Cr(III) DPV1 × 10^−9^ M Cr(VI) 1 × 10^−7^ M Cr(III)	water	[103]
	Cr(VI)-MFC biosensor with *E. aestuarii* YC211	-	-	artificial wastewater electroplating waste-water	[104]
Zn^2+^	Oxytocin (OT)	-	100 fM	healthy and ms sera patients (zinc to copper ions)	[78]
	Oxytocin (OT)	10^−13^–10^−3^ M	-	-	[79]
	Steric paper-based ratio-type	0.1–7000 nM	0.03 nM	water	[105]
	Double-chamber MFC	15–80 mg L^−1^	-	wastewater	[100]
	Microbial fuel cells (MFCs) microalgae and cyanobacteria	2.5–1000 μM	-	ecotoxicology assays	[106]
Tl^+^	Gly modified GCE	2 nM–0.2 mM	0.175 nM	drinking water, spring water, river water, industrial wastewater	[66]
	Tl-IP–MWCNT–CPE	3.0–240 ng mL^−1^	0.76 ng mL^−1^	tap water, well water, wastewater	[107]
	Microbial fuel cells (MFCs) microalgae and cyanobacteria	0.1–3000 μM	-	ecotoxicology assays	[106]

## Abbreviations

Heavy metal ions (HMI), screen-printed electrodes (SPE), artificial functional nucleic acids (FNA), metal-specific oligonucleotides (MSO), artificial deoxyribonucleic acid enzymes (DNAzymes), molecularly imprinted polymers (MIP), limit of detection (LOD), electrogenerated chemiluminescence (ECL), glassy carbon electrode (GCE), pencil graphite electrode (PEG), square wave anodic stripping voltammetry (SWASV), reduced graphene oxide (RGO), gold nanorods (AuNRs), gold nanoparticles (AuNPs), multi-walled carbon nanotubes (MWCNT), thionine (TH), streptavidin (SA), differential pulse voltammetry (DPV), metal-organic frameworks (MOF), cyclic voltammetry (CV), open circuit potential (OCP), differential pulse cathodic stripping voltammetry (DPCSV), composite suspension (CS), carbon black (CB).

## References

[1] Gumpu M.B., Sethuraman S., Krishnan U.M., Rayappan J.B.B. (2015). A review on detection of heavy metal ions in water—An electrochemical approach. Sens. Actuators B Chem..

[2] Zhou Y., Tang L., Zeng G., Zhang C., Zhang Y., Xie X. (2016). Current progress in biosensors for heavy metal ions based on DNAzymes/DNA molecules functionalized nanostructures: A review. Sens. Actuators B Chem..

[3] Wang L., Peng X., Fu H., Huang C., Li Y., Liu Z. (2020). Recent advances in the development of electrochemical aptasensors for detection of heavy metals in food. Biosens. Bioelectron..

[4] De Benedetto G., Di Masi S., Pennetta A., Malitesta C. (2019). Response Surface Methodology for the Optimisation of Electrochemical Biosensors for Heavy Metals Detection. Bionsensors.

[5] Nikolaevna K.A., Svalova T., Malysheva N.N., Okhokhonin A.V., Vidrevich M.B., Brainina K. (2018). Sensors Based on Bio and Biomimetic Receptors in Medical Diagnostic, Environment, and Food Analysis. Bionsensors.

[6] Mehrotra P. (2016). Biosensors and their applications—A review. J. Oral Biol. Craniofacial Res..

[7] Peixoto P.S., Machado A., Oliveira H.P., Bordalo A.A., Segundo M.A. (2019). Paper-Based Biosensors for Analysis of Water. Biosensors for Environmental Monitoring.

[8] Reynoso E.C., Torres E., Bettazzi F., Palchetti I. (2019). Trends and Perspectives in Immunosensors for Determination of Currently-Used Pesticides: The Case of Glyphosate, Organophosphates, and Neonicotinoids. Bionsensors.

[9] Liu X., Yao Y., Ying Y., Ping J. (2019). Recent advances in nanomaterial-enabled screen-printed electrochemical sensors for heavy metal detection. TrAC Trends Anal. Chem..

[10] Ferrari A.G.-M., Carrington P., Rowley-Neale S.J., Banks C.E. (2020). Recent advances in portable heavy metal electrochemical sensing platforms. Environ. Sci. Water Res. Technol..

[11] Moro G., Bottari F., Van Loon J., Du Bois E., De Wael K., Moretto L.M. (2019). Disposable electrodes from waste materials and renewable sources for (bio)electroanalytical applications. Biosens. Bioelectron..

[12] Tchounwou P.B., Yedjou C.G., Patlolla A.K., Sutton D.J. (2012). Molecular, Clinical and Environmental Toxicology. Clinical and Environmental Toxicology. Experientia Supplementum.

[13] Briffa J., Sinagra E., Blundell R. (2020). Heavy metal pollution in the environment and their toxicological effects on humans. Heliyon.

[14] Jaishankar M., Tseten T., Anbalagan N., Mathew B.B., Beeregowda K.N. (2014). Toxicity, mechanism and health effects of some heavy metals. Interdiscip. Toxicol..

[15] Speight J.G., Speight J.G. (2020). 5—Sources of water pollution. Natural Water Remediation.

[16] Council of the European (2020). Union European Drinking Water Directive (DWD) 6230/20 of 7 October 2020 on the Quality of Water Intended for Human Consumption (Recast).

[17] US Environmental Protection Agency (2018). 2018 Edition of the Drinking Water Standards and Health Advisories Tables. https://www.epa.gov/sites/production/files/2018-03/documents/dwtable2018.pdf.

[18] (2010). Council Directive 98/83/EC of 3 November 1998 on the quality of water intended for human consumption (OJ L 330 05.12.1998 p. 32). Doc. Eur. Community Environ. Law.

[19] Lucentini L., Diddi E., Di Martino E.F., Ferretti E., Fuscoletti V., Nigro Di Gregorio F., Veschetti E. (2020). Piani di Sicurezza dell’Acqua nella Gestione di Emergenze Idropotabili: Il Caso del tallio a Pietrasanta e Valdicastello (Lucca). Rapporti ISTISAN 20/8.

[20] WHO (2018). Guidelines for Drinking-Water Quality, 4th Edition, Incorporating the 1st Addendum.

[21] European Union (2010). O.J. of the E. Directive 2010/75/EU of the European Parliament and of the Council of 24 November 2010.

[22] Council of the European Union Commision Regulation (EC) No. 1881/2006. https://eur-lex.europa.eu/legal-content/EN/TXT/PDF/?uri=CELEX:32006R1881&from=IT.

[23] ICH (2019). Guideline for Elemental Impurities Q3D (R1).

[24] Heena, Malik A.K. (2020). Review on Metal Speciation and Their Applications since 2010.

[25] Xing G., Sardar M.R., Lin B., Lin J.-M. (2019). Analysis of trace metals in water samples using NOBIAS chelate resins by HPLC and ICP-MS. Talanta.

[26] Moro G., Barich H., Driesen K., Montiel N.F., Neven L., Mendonça C.D., Shanmugam S.T., Daems E., De Wael K. (2020). Unlocking the full power of electrochemical fingerprinting for on-site sensing applications. Anal. Bioanal. Chem..

[27] Moro G., De Wael K., Moretto L.M. (2019). Challenges in the electrochemical (bio)sensing of nonelectroactive food and environmental contaminants. Curr. Opin. Electrochem..

[28] Moretto L.M., Kalcher K. (2014). Environmental Analysis by Electrochemical Sensors and Biosensors.

[29] Wang R., Wang X. (2020). Sensing of inorganic ions in microfluidic devices. Sens. Actuators B Chem..

[30] Turdean G.L. (2011). Design and Development of Biosensors for the Detection of Heavy Metal Toxicity. Int. J. Electrochem..

[31] Narakathu B., Atashbar M.Z., Bejcek B. (2010). Improved detection limits of toxic biochemical species based on impedance measurements in electrochemical biosensors. Biosens. Bioelectron..

[32] Xie S., Tang Y., Tang D. (2017). Highly sensitive electrochemical detection of mercuric ions based on sequential nucleic acid amplification and guanine nanowire formation. Anal. Methods.

[33] Reddy G.N., Prasad M. (1990). Heavy metal-binding proteins/peptides: Occurrence, structure, synthesis and functions. A review. Environ. Exp. Bot..

[34] Heaton I., Platt M. (2019). Peptide Nanocarriers for Detection of Heavy Metal Ions Using Resistive Pulse Sensing. Anal. Chem..

[35] Janyasupab M., Liu C.-C., Kreysa G., Ota K., Savinell R.F. (2014). Enzymatic Electrochemical Biosensors BT. Encyclopedia of Applied Electrochemistry.

[36] Ashrafi A.M., Sýs M., Sedlackova E., Farag A.S., Adam V., Přibyl J., Richtera L. (2019). Application of the Enzymatic Electrochemical Biosensors for Monitoring Non-Competitive Inhibition of Enzyme Activity by Heavy Metals. Sensors.

[37] Liu J., Cao Z., Lu Y. (2009). Functional Nucleic Acid Sensors. Chem. Rev..

[38] Zhan S., Wu Y., Wang L., Zhan X., Zhou P. (2016). A mini-review on functional nucleic acids-based heavy metal ion detection. Biosens. Bioelectron..

[39] Komarova N., Kuznetsov A. (2019). Inside the Black Box: What Makes SELEX Better?. Molecules.

[40] Sola M., Menon A.P., Moreno B., Meraviglia-Crivelli D., Soldevilla M.M., Cartón-García F., Pastor F. (2020). Aptamers Against Live Targets: Is In Vivo SELEX Finally Coming to the Edge?. Mol. Ther. Nucleic Acids.

[41] Zhuo Z., Yu Y., Wang M., Li J., Zhang Z.-K., Liu J., Wu X., Lu A., Zhang G., Zhang B.-T. (2017). Recent Advances in SELEX Technology and Aptamer Applications in Biomedicine. Int. J. Mol. Sci..

[42] Wang H., Liu Y., Liu G. (2018). Reusable resistive aptasensor for Pb (II) based on the Pb(II)-induced despiralization of a DNA duplex and formation of a G-quadruplex. Microchim. Acta.

[43] Farzinb L., Shamsipur M., Sheibani S. (2017). A review: Aptamer-based analytical strategies using the nanomaterials for environmental and human monitoring of toxic heavy metals. Talanta.

[44] Díaz-Amaya S., Lin L.-K., DiNino R.E., Ostos C., Stanciu L.A. (2019). Inkjet printed electrochemical aptasensor for detection of Hg^2+^ in organic solvents. Electrochim. Acta.

[45] Dairaku T., Furuita K., Sato H., Šebera J., Yamanaka D., Otaki H., Kikkawa S., Kondo Y., Katahira R., Bickelhaupt F.M. (2015). Direct detection of the mercury–nitrogen bond in the thymine–HgII–thymine base-pair with 199Hg NMR spectroscopy. Chem. Commun..

[46] Liu G., Li Z., Zhu J., Liu Y., Zhou Y., He J. (2015). Studies on the thymine–mercury–thymine base pairing in parallel and anti-parallel DNA duplexes. New J. Chem..

[47] Yu S.H., Kim T.H. (2019). T-T Mismatch-Based Electrochemical Aptasensor for Ultratrace Level Detection of Hg^2+^ Using Electrochemically Reduced Graphene Oxide-Modified Electrode. J. Biomed. Nanotechnol..

[48] Lu Z., Wang P., Xiong W., Qi B., Shi R., Xiang D., Zhai K. (2020). Simultaneous detection of mercury (II), lead (II) and silver (I) based on fluorescently labelled aptamer probes and graphene oxide. Environ. Technol..

[49] Dolati S., Ramezani M., Abnous K., Taghdisi S.M. (2017). Recent nucleic acid based biosensors for Pb^2+^ detection. Sens. Actuators B Chem..

[50] Cui H., Xiong X., Gao B., Chen Z., Luo Y., He F., Deng S., Chen L. (2016). A Novel Impedimetric Biosensor for Detection of Lead (II) with Low-cost Interdigitated Electrodes Made on PCB. Electroanalytical.

[51] Han S., Wang R., Tang Y., He M., Zhang X., Shi H., Xiang Y. (2016). Practical, highly sensitive, and regenerable evanescent-wave biosensor for detection of Hg^2+^ and Pb^2+^ in water. Biosens. Bioelectron..

[52] Perera R., Ashraf S., Mueller A. (2017). The binding of metal ions to molecularly-imprinted polymers. Water Sci. Technol..

[53] Malitesta C., Di Masi S., Mazzotta E. (2017). From Electrochemical Biosensors to Biomimetic Sensors Based on Molecularly Imprinted Polymers in Environmental Determination of Heavy Metals. Front. Chem..

[54] An Z., Liu W., Liang Q., Yan G., Qin L., Chen L., Wang M., Yang Y., Liu X. (2018). Ion-Imprinted Polymers Modified Sensor for Electrochemical Detection of Cu^2+^. Nano.

[55] Branger C., Meouche W., Margaillan A. (2013). Recent advances on ion-imprinted polymers. React. Funct. Polym..

[56] Florea A., Feier B., Cristea C., Marć M. (2019). Chapter Eight—In Situ Analysis Based on Molecularly Imprinted Polymer Electrochemical Sensors. Mip Synthesis, Characteristics and Analytical Application.

[57] Gui R., Guo H., Jin H. (2019). Preparation and applications of electrochemical chemosensors based on carbon-nanomaterial-modified molecularly imprinted polymers. Nanoscale Adv..

[58] Crapnell R., Hudson A., Foster C.W., Eersels K., Van Grinsven B., Cleij T.J., Banks C.E., Peeters M. (2019). Recent Advances in Electrosynthesized Molecularly Imprinted Polymer Sensing Platforms for Bioanalyte Detection. Sensors.

[59] Rico-Yuste A., Carrasco S. (2019). Molecularly Imprinted Polymer-Based Hybrid Materials for the Development of Optical Sensors. Polymers.

[60] Bala A., Górski Ł. (2018). Peptide nucleic acid as a selective recognition element for electrochemical determination of Hg^2+^. Bioelectrochemistry.

[61] Zhang X., Zhu M., Jiang Y., Wang X., Guo Z., Shi J., Zou X., Han E. (2020). Simple electrochemical sensing for mercury ions in dairy product using optimal Cu^2+^-based metal-organic frameworks as signal reporting. J. Hazard. Mater..

[62] Cheng L., Wei B., He L.L., Mao L., Zhang J., Ceng J., Kong D., Chen C., Cui H., Hong N. (2017). “Off-On” switching electrochemiluminescence biosensor for mercury(II) detection based on molecular recognition technology. Anal. Biochem..

[63] Bala A., Górski Ł. (2016). Determination of mercury cation using electrode modified with phosphorothioate oligonucleotide. Sens. Actuators B Chem..

[64] Jin H., Zhang M., Wei M., Cheng J. (2019). A voltammetric biosensor for mercury(II) using reduced graphene oxide@gold nanorods and thymine-Hg(II)-thymine interaction. Microchim. Acta.

[65] Yaddaden C., Benamar M., Gabouze N., Berouaken M., Ayat M. (2019). Investigations on mercury ion detection in aqueous solution by triglycine surface activated porous silicon nanowires. Phys. E Low Dimens. Syst. Nanostruct..

[66] Shah A., Nisar A., Khan K., Nisar J., Niaz A., Ashiq M.N., Akhter M.S. (2019). Amino acid functionalized glassy carbon electrode for the simultaneous detection of thallium and mercuric ions. Electrochim. Acta.

[67] Xie H., Wang Q., Chai Y., Yuan Y., Yuan R. (2016). Enzyme-assisted cycling amplification and DNA-templated in-situ deposition of silver nanoparticles for the sensitive electrochemical detection of Hg^2+^. Biosens. Bioelectron..

[68] Wang H., Liu Y., Wang J., Xiong B., Hou X. (2020). Electrochemical impedance biosensor array based on DNAzyme-functionalized single-walled carbon nanotubes using Gaussian process regression for Cu(II) and Hg(II) determination. Microchim. Acta.

[69] Zhang X., Jianga Y., Zhua M., Xua Y., Guoa Z., Shi J., Hana E., Zou X., Wangb D. (2020). Electrochemical DNA sensor for inorganic mercury(II) ion at attomolar level in dairy product using Cu(II)-anchored metal-organic framework as mimetic catalyst. Chem. Eng. J..

[70] Mohammadi A., Heydari-Bafrooei E., Foroughi M.M., Mohammadi M. (2020). Heterostructured Au/MoS2-MWCNT nanoflowers: A highly efficient support for the electrochemical aptasensing of solvated mercuric ion. Microchem. J..

[71] Xie H., Niu Y., Deng Y., Cheng H., Ruan C., Li G., Sun W. (2020). Electrochemical aptamer sensor for highly sensitive detection of mercury ion with Au/Pt@carbon nanofiber-modified electrode. J. Chin. Chem. Soc..

[72] Ma F., Chen Y., Zhu Y., Liu J. (2019). Electrogenerated chemiluminescence biosensor for detection of mercury (II) ion via target-triggered manipulation of DNA three-way junctions. Talanta.

[73] Hasanjani H.R.A., Zarei K. (2019). An electrochemical sensor for attomolar determination of mercury(II) using DNA/poly-L-methionine-gold nanoparticles/pencil graphite electrode. Biosens. Bioelectron..

[74] Tang W., Wang Z., Yu J., Zhang F., He P.-G. (2018). Internal Calibration Potentiometric Aptasensors for Simultaneous Detection of Hg^2+^, Cd^2+^, and As^3+^ Based on a Screen-Printed Carbon Electrodes Array. Analyst Chem..

[75] Pérez-Ràfols C., Serrano N., Díaz-Cruz J.M., Ariño C., Esteban M. (2016). Glutathione modified screen-printed carbon nanofiber electrode for the voltammetric determination of metal ions in natural samples. Talanta.

[76] Yu Y., Yu C., Yin T., Ou S., Sun X., Wen X., Zhang L., Tang D., Yin X.-X. (2017). Functionalized poly (ionic liquid) as the support to construct a ratiometric electrochemical biosensor for the selective determination of copper ions in AD rats. Biosens. Bioelectron..

[77] Yin X., Wang P., Zhu X., Peng Q., Zhou Y., Yin T., Liang Y., Yin X. (2018). Combined determination of copper ions and β-amyloid peptide by a single ratiometric electrochemical biosensor. Analyst.

[78] Tadi K.K., Alshanski I., Mervinetsky E., Marx G., Petrou P., Dimitrios K.M., Gilon C., Hurevich M., Yitzchaik S. (2017). Oxytocin-Monolayer-Based Impedimetric Biosensor for Zinc and Copper Ions. ACS Omega.

[79] Mervinetsky E., Alshanski I., Buchwald J., Dianat A., Lončarić I., Lazić P., Crljen Ž., Gutierrez R., Cuniberti G., Hurevich M. (2019). Direct Assembly and Metal-Ion Binding Properties of Oxytocin Monolayer on Gold Surfaces. Langmuir.

[80] Tian R., Chen X., Liu D., Yao C. (2016). A Sensitive Biosensor for Determination of Cu^2+^ by One-step Electrodeposition. Electroanalytical.

[81] Wei P., Zhu Z., Song R., Li Z., Chen C. (2019). An ion-imprinted sensor based on chitosan-graphene oxide composite polymer modified glassy carbon electrode for environmental sensing application. Electrochim. Acta.

[82] Li J., Zhang L., Wei G., Zhang Y., Zeng Y. (2015). Highly sensitive and doubly orientated selective molecularly imprinted electrochemical sensor for Cu^2+^. Biosens. Bioelectron..

[83] Qiu B., Qiu J., Cui M., Wei X., Zhao M., Qiu B., Chen G. (2016). An immobilization free DNAzyme based electrochemical biosensor for lead determination. Analyst.

[84] Wang L., Wen Y., Li L., Yang X., Jia N., Li W., Meng J., Duan M., Sun X., Liu G. (2018). Sensitive and label-free electrochemical lead ion biosensor based on a DNAzyme triggered G-quadruplex/hemin conformation. Biosens. Bioelectron..

[85] Sebastian M., Mathew B. (2018). Ion imprinting approach for the fabrication of an electrochemical sensor and sorbent for lead ions in real samples using modified multiwalled carbon nanotubes. J. Mater. Sci..

[86] Alizadeh T., Hamidi N., Ganjali M.R., Rafiei F. (2017). An extraordinarily sensitive voltammetric sensor with picomolar detection limit for Pb 2+ determination based on carbon paste electrode impregnated with nano-sized imprinted polymer and multi-walled carbon nanotubes. J. Environ. Chem. Eng..

[87] Ghanei-Motlagh M., Taher M.A. (2017). An Electrochemical Sensor Based on Novel Ion Imprinted Polymeric Nanoparticles for Selective Detection of Lead Ions. Anal. Bioanal. Chem. Res..

[88] Dahaghin Z., Kilmartin P.A., Mousavi H.Z. (2020). Novel ion imprinted polymer electrochemical sensor for the selective detection of lead (II). Food Chem..

[89] Dali M., Zinoubi K., Chrouda A., Abderrahmane S., Cherrad S., Jaffrezic-Renault N. (2018). A biosensor based on fungal soil biomass for electrochemical detection of lead (II) and cadmium (II) by differential pulse anodic stripping voltammetry. J. Electroanal. Chem..

[90] Florescu M., David M., Badea M. (2018). Development And Evaluation of Sol-Gel-Based Biosensors for Cadmium Ions Detection. Environ. Eng. Manag. J..

[91] Fourou H., Zazoua A., Braiek M., Jaffrezic-Renault N. (2016). An enzyme biosensor based on beta-galactosidase inhibition for electrochemical detection of cadmium (II) and chromium (VI). Int. J. Environ. Anal. Chem..

[92] Gumpu M.B., Veerapandian M., Krishnan U.M., Rayappan J.B.B. (2018). Amperometric determination of As(III) and Cd(II) using a platinum electrode modified with acetylcholinesterase, ruthenium(II)-tris(bipyridine) and graphene oxide. Microchim. Acta.

[93] Zhad H.R.L.Z., Torres Y.M.R., Lai R.Y. (2017). A reagentless and reusable electrochemical aptamer-based sensor for rapid detection of Cd (II). J. Electroanal. Chem..

[94] Li Y., Ran G., Lu G., Ni X., Liu D., Sun J., Xie C., Yao D., Bai W. (2019). Highly Sensitive Label-Free Electrochemical Aptasensor Based on Screen-Printed Electrode for Detection of Cadmium (II) Ions. J. Electrochem. Soc..

[95] Fakude C.T., Arotiba O., Mabuba N. (2020). Electrochemical aptasensing of cadmium (II) on a carbon black-gold nano-platform. J. Electroanal. Chem..

[96] Liu Y., Lai Y., Yang G., Tang C., Deng Y., Li S., Wang Z. (2017). Cd-Aptamer Electrochemical Biosensor Based on AuNPs/CS Modified Glass Carbon Electrode. J. Biomed. Nanotechnol..

[97] Wang X., Gao W., Yan W., Li P., Zou H., Wei Z., Guan W., Ma Y., Zou H., Wenyu G. (2018). A Novel Aptasensor Based on Graphene/Graphite Carbon Nitride Nanocomposites for Cadmium Detection with High Selectivity and Sensitivity. ACS Appl. Nano Mater..

[98] Aravind A., Mathew B. (2018). Tailoring of nanostructured material as an electrochemical sensor and sorbent for toxic Cd(II) ions from various real samples. J. Anal. Sci. Technol..

[99] Hu S. (2019). An Electrochemical Sensor Based on ion Imprinted PPy/rGO Composite for Cd(II) Determination in Water. Int. J. Electrochem. Sci..

[100] Xie T., Gao Y.-M., Zheng Q., Rt H.-, Wang X.-H., Li Y., Luo N., Liu R. (2017). A Double-microbial Fuel Cell Heavy Metals Toxicity Sensor. DEStech Trans. Environ. Energy Earth Sci..

[101] Behrmann I.C.L., Grattieri M., Minteer S.D., Ramirez S.A., Vullo D.L. (2020). Online self-powered Cr(VI) monitoring with autochthonous Pseudomonas and a bio-inspired redox polymer. Anal. Bioanal. Chem..

[102] Garavaglia L., Cerdeira S.B., Vullo D.L. (2010). Chromium (VI) biotransformation by β- and γ-Proteobacteria from natural polluted environments: A combined biological and chemical treatment for industrial wastes. J. Hazard. Mater..

[103] Prabhakaran D.C., Riotte J., Sivry Y., Subramanian S. (2017). Electroanalytical Detection of Cr(VI) and Cr(III) Ions Using a Novel Microbial Sensor. Electroanalytical.

[104] Wu L.-C., Tsai T.-H., Liu M.-H., Kuo J.-L., Chang Y.-C., Chung Y.-C. (2017). A Green Microbial Fuel Cell-Based Biosensor for In Situ Chromium (VI) Measurement in Electroplating Wastewater. Sensors.

[105] Li L., Zhang Y., Zhang L., Ge S., Yan M., Yu J. (2017). Steric paper based ratio-type electrochemical biosensor with hollow-channel for sensitive detection of Zn^2+^. Sci. Bull..

[106] Labro J., Craig T., Wood S.A., Packer M. (2017). Demonstration of the use of a photosynthetic microbial fuel cell as an environmental biosensor. Int. J. Nanotechnol..

[107] Nasiri-Majd M., Taher M.A., Fazelirad H. (2015). Synthesis and application of nano-sized ionic imprinted polymer for the selective voltammetric determination of thallium. Talanta.

[108] Marnane I., Jeroen K., Carlijn H., Visschedijk A., Tycho S., Vermeulen J., Grandjean P., Hagström P., Per K., Fold N. (2018). EU Publications Mercury in Europe’s Environment a Priority for European and Global Action—1977-8449EEA Report No 11/2018.

[109] Selin N.E. (2009). Global Biogeochemical Cycling of Mercury: A Review. Annu. Rev. Environ. Resour..

[110] Streets D.G., Horowitz H.M., Jacob D.J., Lu Z., Levin L., Ter Schure A.F.H., Sunderland E.M. (2017). Total Mercury Released to the Environment by Human Activities. Environ. Sci. Technol..

[111] Park J.-D., Zheng W. (2012). Human Exposure and Health Effects of Inorganic and Elemental Mercury. J. Prev. Med. Public Health.

[112] Bernhoft R.A. (2011). Mercury Toxicity and Treatment: A Review of the Literature. J. Environ. Public Health.

[113] Hong Y.-S., Kim Y.-M., Lee K.-E. (2012). Methylmercury Exposure and Health Effects. J. Prev. Med. Public Health.

[114] Genchi G., Sinicropi M.S., Carocci A., Lauria G., Catalano A. (2017). Mercury Exposure and Heart Diseases. Int. J. Environ. Res. Public Health.

[115] Bose-O’Reilly S., Mccarty K.M., Steckling N., Lettmeier B. (2010). Mercury Exposure and Children’s Health. Curr. Probl. Pediatr. Adolesc. Health Care.

[116] Liu T., Chu Z., Jin W. (2019). Electrochemical mercury biosensors based on advanced nanomaterials. J. Mater. Chem. B.

[117] Kokkinos C., Economou A., Pournara A., Manos M., Spanopoulos I., Kanatzidis M., Tziotzi T., Petkov V., Margariti A., Oikonomopoulos P. (2020). 3D-printed lab-in-a-syringe voltammetric cell based on a working electrode modified with a highly efficient Ca-MOF sorbent for the determination of Hg(II). Sens. Actuators B Chem..

[118] Desai V., Kaler S.G. (2008). Role of copper in human neurological disorders. Am. J. Clin. Nutr..

[119] Witt B., Schaumlöffel D., Schwerdtle T. (2020). Subcellular Localization of Copper—Cellular Bioimaging with Focus on Neurological Disorders. Int. J. Mol. Sci..

[120] Gaetke L.M. (2003). Copper toxicity, oxidative stress, and antioxidant nutrients. Toxicology.

[121] Barceloux D.G. (1999). Copper. J. Toxicol. Clin. Toxicol..

[122] Li F., Ma W., Liu J., Wu X., Wang Y., He J. (2018). Luminol, horseradish peroxidase, and glucose oxidase ternary functionalized graphene oxide for ultrasensitive glucose sensing. Anal. Bioanal. Chem..

[123] Grant L.D. (2009). Lead and Compounds. Environ. Toxic..

[124] Rani A., Kumar A., Lal A., Pant M. (2014). Cellular mechanisms of cadmium-induced toxicity: A review. Int. J. Environ. Health Res..

[125] Godt J.C., Scheidig F., Grosse-Siestrup C., Esche V., Brandenburg P., Reich A., A Groneberg D. (2006). The toxicity of cadmium and resulting hazards for human health. J. Occup. Med. Toxicol..

[126] Huff J., Lunn R.M., Waalkes M.P., Tomatis L., Infante P.F. (2007). Cadmium-induced Cancers in Animals and in Humans. Int. J. Occup. Environ. Health.

[127] Zhou T., Han H., Liu P., Xiong J., Tian F., Li X. (2017). Microbial Fuels Cell-Based Biosensor for Toxicity Detection: A Review. Sensors.

[128] Yu D., Bai L., Zhai J., Wang Y., Dong S. (2017). Toxicity detection in water containing heavy metal ions with a self-powered microbial fuel cell-based biosensor. Talanta.

[129] Imai T., Okamura H. (1994). Study on Incineration Method of Leather Scraps and Recovery of Chromium from Incinerated Residues.

[130] Frassinetti S., Bronzetti G.L., Caltavuturo L., Cini M., Della Croce C. (2006). The Role of Zinc in Life: A Review. J. Environ. Pathol. Toxicol. Oncol..

[131] Plum L.M., Rink L., Haase H. (2010). The Essential Toxin: Impact of Zinc on Human Health. Int. J. Environ. Res. Public Health.

[132] Wedepohl K.H. (1995). The composition of the continental crust. Geochim. Cosmochim. Acta.

[133] Riley J., Siddiqui S. (1986). The determination of thallium in sediments and natural waters. Anal. Chim. Acta.

[134] Zhang C., Ren S., Cheng H., Zhang W., Ma J., Zhang C., Guo Z. (2018). Thallium pollution and potential ecological risk in the vicinity of coal mines in Henan Province, China. Chem. Speciat. Bioavailab..

